# Reducing Public Stigma Toward Suicide‐Loss Survivors Through Brief Video Interventions: A Randomized Controlled Trial

**DOI:** 10.1155/da/2062450

**Published:** 2026-07-20

**Authors:** Yossi Levi-Belz, Chana T. Fisch, Amsalem Doron

**Affiliations:** ^1^ The Lior Tsfaty Center for Suicide and Mental Pain Studies, University of Haifa, Haifa, Israel, haifa.ac.il; ^2^ School of Therapy, Counseling and Human Development, University of Haifa, Haifa, Israel, haifa.ac.il; ^3^ New York State Psychiatric Institute and Department of Psychiatry, New York, New York, USA; ^4^ Columbia University Vagelos College of Physicians and Surgeons, New York, New York, USA, vagelos.columbia.edu

**Keywords:** contact-based video intervention, postvention, public stigma, randomized controlled trial, suicide bereavement

## Abstract

**Background:**

Suicide‐loss survivors (SLSs) experience substantial and often enduring psychological burden. These difficulties are compounded by public stigma, underscoring the need for scalable approaches to shift attitudes at the population level. In this study, we examined whether brief survivor‐narrative videos can reduce public stigma of SLSs.

**Methods:**

In a randomized controlled trial, 1351 adults (18–50) completed baseline measures and were allocated to view either a brief SLS narrative or a psychoeducational control. Public stigma toward SLSs and trait impressions were assessed at baseline and postexposure.

**Results:**

Relative to control, the SLS video arm showed clear improvements immediately and at 30 days: stigma scores were lower and trait impressions more favorable, with attenuation over time. Item‐level analyses indicated sizable immediate reductions for “Disconnected” (−24%) and “Cowardly” (−16%), and smaller but significant decreases for “Immoral” and “Irresponsible” (−12% each).

**Conclusions:**

Brief survivor‐narrative videos can shift public attitudes toward SLSs and maintain part of that change over 1 month. As a low‐cost, scalable complement to postvention, brief video contact offers a practical lever to improve the social climate surrounding SLSs. Deployed widely and reinforced over time, it can move communities from blame to empathy, strengthen everyday support, and advance survivors’ recovery.

**Summary**

• Suicide bereavement is known to be associated with elevated risks for depression, PTSD, and suicidality; little research has addressed the unique role of public stigma in compounding these vulnerabilities. This study fills that gap by testing a brief, scalable intervention aimed at reducing stigma at the population level.
• When supporting suicide‐loss survivors (SLSs), clinicians should attend not only to symptoms of grief and trauma but also to the social context of stigma, as negative societal attitudes can intensify distress, hinder help‐seeking, and obstruct recovery.
• Mental health practitioners, educators, and policymakers should recognize the potential of survivor‐narrative video interventions as low‐cost tools to shift public attitudes, foster empathy, and strengthen the everyday social support available to bereaved families.

## 1. Introduction

Suicide bereavement constitutes a unique and devastating form of loss that extends far beyond the immediate tragedy of death [1, 2]. Epidemiological estimates suggest that each suicide directly impacts five close relatives and up to 135 members of the broader community, resulting in nearly 60 million new suicide‐loss survivors (SLSs) worldwide every year [3].

Compared to other bereavement groups, SLSs are consistently found to experience elevated risks for depression, posttraumatic stress, suicidal ideation, suicidal behavior, and negative physical health outcomes [2, 4–7]. In addition, SLSs frequently struggle with complicated or prolonged grief, characterized by persistent yearning, intrusive counterfactual thinking, and difficulties in meaning‐making that can last for years after the death [8, 9].

The psychological toll is also compounded by intense self‐directed emotions—such as guilt, shame, and anger—as well as the existential struggle to comprehend the self‐inflicted nature of the death [10, 11]. These cognitive and emotional burdens [12] not only intensify individual suffering but also destabilize family systems and erode social bonds, thereby amplifying the intergenerational and community‐level impact of suicide bereavement [13]. These compounded vulnerabilities highlight suicide bereavement not merely as an individual tragedy but as a pressing public health issue that demands dedicated scientific and clinical attention.

### 1.1. Stigma in Mental Health and Suicide Bereavement

Stigma has long been recognized as a central social determinant that shapes the course of mental illness and recovery. Conceptually, it comprises processes of labeling, stereotyping, and discrimination that operate at multiple levels; public stigma, in particular, reflects society’s endorsement of negative stereotypes about people affected by a mental illness [14, 15]. Public stigma reduces the willingness to interact with affected individuals and directly undermines help‐seeking and treatment engagement [16]. Taken together, these mechanisms suggest that stigma may function as a substantial, population‐level obstacle to recovery [17].

Beyond the inherent psychological challenges of suicide bereavement, SLSs are uniquely burdened by the pervasive presence of stigma, which can substantially exacerbate the difficulties they face [18]. Accumulating evidence shows that, compared with other bereaved groups, SLSs are more likely to encounter judgment, blame, social avoidance, and rejection [15, 19]. Unlike the stigma of suicide itself, in which the focus is the individual who has attempted or died by suicide, SLSs are often judged for presumed moral failure, experience social withdrawal by others, and internalize shame and guilt [1, 19]. A higher perceived stigma is associated with prolonged/complicated grief, major depression, and elevated suicidal ideation [18]. Importantly, prolonged grief itself can heighten stigmatizing responses from others, thereby reinforcing a bidirectional, self‐perpetuating cycle [8]. Thus, stigma not only compounds the psychological burden of suicide bereavement but also fuels a vicious spiral in which distress and stigmatization intensify one another, further isolating survivors and obstructing recovery [20].

### 1.2. Efforts to Reduce Public Stigma

Given its pervasive impact, considerable research has focused on identifying effective strategies to reduce the mental health stigma. While educational approaches can correct factual misconceptions, meta‐analyses suggest that social contact‐based interventions—exposure to personal narratives from individuals with lived experience—are more powerful in shifting public attitudes [21]. Specifically, brief video‐based contact interventions have shown substantial promise. In a series of studies, Amsalem and colleagues demonstrated that brief videos reduce stigma across conditions, including psychosis and depression, with effects lasting from weeks to months [22, 23]. However, there remains a striking absence of evidence regarding interventions to address the stigma toward SLSs. This gap is critical. Public narratives frequently ascribe responsibility, weakness, or “contamination” to families bereaved by suicide, reinforcing cycles of shame and silence [24]. Because stigma manifests most potently in the social arena—through avoidance, gossip, and rejection—brief, social contact‐based interventions that leverage compelling first‐person narratives may be especially positioned to shift public perceptions.

Translating this approach to suicide bereavement, survivors’ testimonies in a video format could explicitly counter myths of blame, model adaptive coping, and emphasize the enduring humanity and resilience of bereaved families. Such interventions have the potential not only to improve societal attitudes but also to enhance the availability of social support and thereby mitigate secondary psychological harm among SLSs. However, to date, no studies have examined whether brief video interventions can reduce public stigma toward SLSs. Evaluating the potential of such a low‐cost and scalable approach to influence public attitudes represents an important and timely step.

### 1.3. The Present Study

The present study aimed to address these gaps by testing the effectiveness of brief video interventions featuring different SLSs—a father, a mother, and a sibling of a person who died by suicide, sharing their lived experiences of bereavement—in reducing stigmatizing attitudes and improving public perceptions. Specifically, in this study, we compared participants exposed to one of these SLS videos with those exposed to a written psychoeducational control. This design allowed us to examine whether narratives of lived experience offer unique benefits beyond informational content alone. We hypothesized that, relative to the control condition, exposure to SLS videos would lead to the following:

1. Reductions in stigmatizing attitudes toward SLSs, reflected in lower endorsement of negative stereotypes such as blame and dysfunction.
2. Trait impressions of SLSs would improve, with participants perceiving them as warmer and more competent.
3. These effects would be sustained over time, such that reductions in stigma and improvements in impressions would remain detectable at the 1‐month follow‐up, albeit with some attenuation in magnitude relative to the immediate postexposure assessment.

By testing these hypotheses, the current study seeks to offer preliminary evidence on whether brief video narratives can influence public stigma toward SLSs, potentially informing future research and practice in suicide postvention.

## 2. Methods

### 2.1. Participants and Recruitment

We recruited Hebrew‐speaking Israeli adults between the ages of 18 and 50 using Panel4All, an established online panel commonly used in mental health research in Israel. Recruitment occurred during June and July 2025. The sample size was determined based on power analyses conducted using effect sizes observed in our previous, similar studies. The selected age range was informed by prior studies indicating that younger and middle‐aged adults are generally more responsive to short‐form video content and more likely to engage with digital interventions [25–27]. This population tends to be more familiar with online formats and more open to psychologically oriented media delivered in concise, narrative formats [28, 29]. The Panel4All platform provides access to a diverse sample and includes built‐in features to detect bots, ensure demographic consistency, and block duplicate participation. Participants received modest compensation through the platform. The study protocol was approved by the Ethics Committee of the Ruppin Academic Center.

### 2.2. Ethical Considerations and Procedures

The study received approval from the Institutional Ethics Committee of Ruppin Academic Center (Protocol 167/2025). All procedures adhered to the Declaration of Helsinki. Participants were provided with detailed written information and gave full informed consent electronically prior to participation.

After providing informed consent, participants completed the baseline assessment and were randomly assigned to one of two conditions: the SLS video intervention or the written psychoeducational control. Randomization was implemented through the Qualtrics platform using automated allocation. After viewing the assigned video, participants completed a postintervention assessment and were recontacted for a 30‐day follow‐up. To ensure data quality, attention checks were embedded throughout the survey, and participants were required to spend a minimum amount of time on each stimulus before progressing. Duplicate entries and invalid responses were excluded prior to the analysis.

#### 2.2.1. Video Intervention and Control

Participants assigned to the intervention group viewed one of three brief videos, ranging from 2 min and 29 s to 2 min and 46 s in length. All videos followed a consistent narrative structure and thematic focus. Each featured a first‐person account by an actor portraying a family member—a mother, a father, or a brother—who had lost a loved one to suicide. Although the protagonists were actors, this approach was used to ensure standardization of delivery and to protect the privacy of individuals with a lived experience. Importantly, prior work from our group has demonstrated that actor‐delivered narratives are comparable in effectiveness to those delivered by individuals with lived experience, provided that the stories are authentic [30]. Accordingly, all scripts were grounded in real experiences and informed by a qualitative survey completed by 80 SLSs, allowing individuals to share their stories without broad personal exposure while preserving the core principles of contact‐based interventions. The narrators described their experiences of grief, guilt, and isolation, as well as the social stigma and self‐blame they encountered following the loss. Common themes across the videos included questioning whether more could have been done, the burden of silence, and the fear of being judged by others. While there are a range of accepted models for understanding the root causes of depression, this intervention was grounded in a biomedical one, such that each narrator emphasized that depression is a serious and sometimes fatal illness—not a reflection of family failure—and highlighted that even strong, supportive families can lose a loved one to suicide. This conceptualization is useful for stigma interventions, as it removes individual blame. Each narrator described a shift from silence to sharing—through conversations with friends, family, or support groups—which brought emotional relief and reduced feelings of shame. The videos aimed to foster empathy, reduce stigma toward suicide‐bereaved families, and promote help‐seeking. Video links and full scripts are provided in Supporting Information 1: Supplement 2.

The control group was provided with descriptive psychoeducational text explaining stigma toward SLSs without any personal narrative or emotional content. The text provided an overview of stigma in the realm of suicide, including its role as a barrier to treatment and open conversation and the impact it has on family members. The psychoeducational text is provided in Supporting Information 1: Supplement 2.

### 2.3. Measures

The primary outcomes were perceptions of character traits associated with SLSs, measured through ratings of both negative and positive attributes and self‐reported stigma toward SLSs, defined here as family members of individuals who died by suicide.

#### 2.3.1. Character Trait Ratings

Perceptions of SLSs were assessed by asking participants to rate the extent to which 16 traits described a person whose family member had died by suicide [24]. The list included 12 negative traits (isolated, anxious, irritable, impulsive, emotionally unstable, irresponsible, weak, guilty, cold, secretive, fragile, and pathetic) and 4 positive traits (strong, devoted, brave, and authentic). Ratings were provided on a 5‐point Likert scale from 1 (not at all descriptive) to 5 (very descriptive). Items were analyzed as separate negative and positive subscales. Cronbach’s alpha for this study was 0.87.

#### 2.3.2. Suicide Stigma Scale

Stigmatizing attitudes toward suicide‐bereaved families were measured using a 16‐item scale developed for this study. Each item reflected a common stereotype or stigmatizing belief, such as, “When someone in the family dies by suicide, it means something is wrong with the family” and “If the family had been more attentive, they could have saved the person who died by suicide.” Participants rated their agreement with each item on a 5‐point Likert scale from 1 (strongly disagree) to 5 (strongly agree). Higher scores indicated a greater endorsement of stigmatizing beliefs. Cronbach’s alpha for this study was 0.90.

All measures were administered at three time points: baseline, immediately after the intervention, and at a 30‐day follow‐up. Demographic data collected at baseline included age, gender, ethnicity, income, and level of education.

### 2.4. Statistical Analysis

Analyses were conducted using IBM SPSS Statistics Version 29. Group differences at baseline were assessed using independent *t*‐tests and chi‐square tests, as appropriate. Intervention effects were analyzed using generalized estimating equations (GEEs), which allow for repeated measures and account for missing data through population‐averaged estimates [31, 32]. Models included main effects of group and time and their interactions. Between‐group effect sizes were reported using Cohen’s *d*, and significance was defined as *p* < 0.05 (two‐sided). For item‐level analysis of stigmatizing perceptions, responses were dichotomized into stigmatizing vs. nonstigmatizing views. We then calculated the proportion of participants who shifted from stigmatizing to nonstigmatizing responses postintervention and at follow‐up. McNemar’s test was used to assess within‐subject changes across time points for each item.

## 3. Results

### 3.1. Sample Characteristics

After the exclusion of 187 (12%) participants who failed validity tests (duplications, attention checks, or were identified as bots), our final sample included 1351 individuals who completed the baseline assessments and were randomized into study groups. Of those, 1294 (96%) participants completed the postintervention assessment, and 1040 (77%) completed the 30‐day follow‐up assessment (Supporting Information 2: Supplement 1). As can be seen on Table 1, demographic characteristics did not differ between study groups, and baseline characteristics did not differ between completers and noncompleters. The ± SD respondent age was 33.4 ± 8.6 (range 18–50), and more than half of participants were female (*n* = 765, 57%). Five participants were transgender or nonbinary.

**Table 1:** Demographic characteristics.

Items	Intervention (*n* = 1012)		Controln (*n* = 339)		Total (*N* = 1351)		Statistic	
	Mean	SD	Mean	SD	Mean	SD	Stat^a^	*p*
Age (range 18–50)	33.1	8.7	34.3	8.4	33.4	8.6	5.4	0.021
	***n***	**%**	***n***	**%**	***n***	**%**	***X* ^2^**	***p***
Gender							1.7	0.641
Woman	572	57	193	57	765	57		
Man	435	43	146	43	581	43		
Transgender/nonbinary/other	5	0	0	0	5	0		
Ethnicity							0.28	0.871
Jewish	851	84	284	84	1135	84		
Arab	136	13	48	14	184	14		
Other^b^	25	3	7	2	32	2		
Education							1.5	0.833
Never completed high school	33	3	7	2	40	3		
High school graduate	264	26	92	27	356	26		
Some college credit	227	22	79	23	306	23		
Bachelor’s degree or higher	488	48	161	47	632	47		
Income							7.6	0.108
Significantly below average	270	27	67	20	337	25		
Below average	213	21	86	25	299	22		
Average	308	30	112	33	420	31		
Above average	187	19	62	18	249	18		
Significantly above average	34	3	12	4	46	3		
Familiarity with a person who died by suicide							1.8	0.608
Immediate family	41	4	9	3	50	4		
Close friend or extended family	176	17	63	19	239	18		
Acquaintance	411	41	134	40	545	40		
No Familiarity	414	41	139	41	553	41		

*Note:* Pearson chi‐square.

^a^One‐way ANOVA.

^b^Other: Druze (*n* = 9), Filipino (*n* = 1), Christian (*n* = 4), unspecified (*n* = 18).

### 3.2. Intervention Effects

**Figure 1:**
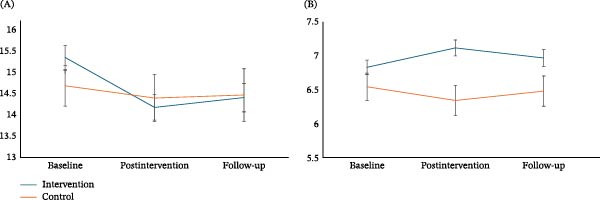
Changes in characteristic impressions by study arm over time. (A) Negative characteristics. (B) Positive characteristics.

As hypothesized, study group outcomes significantly differed on character ratings (negative and positive). Figure 1 presents the GEE model results. Groups did not significantly differ at baseline for negative character ratings (video intervention: 15.3 ± 9.3 (mean ± SD); psychoeducational control: 14.7 ± 8.8; *t* = 1.1, *p* = 0.25) or positive character ratings (video intervention: 6.8 ± 3.2; control: 6.5 ± 3.4; *t* = 1.3, *p* = 0.18). A significant group‐by‐time interaction emerged for negative character ratings (*χ* ^2^ = 8.6, df = 2, *p* = 0.014). From baseline to postintervention, the video group showed a greater reduction in negative character ratings (mean change = 1.2, 95% CI: 0.8–1.5; Cohen’s *d* = 0.13) compared to the control group (mean change = 0.2, 95% CI: –0.4 to 0.8; *d* = 0.02; *p* < 0.001). Effects persisted at the 30‐day follow‐up, with a reduction of 0.8 points (95% CI: 0.3–1.4; *d* = 0.08) in the video group compared to 0.2 (95% CI: –1.0 to 1.4; *d* = 0.02) in the control group (*p* = 0.005). For positive character ratings, a significant group‐by‐time interaction also emerged (*χ* ^2^ = 9.9, df = 2, *p* = 0.007). From baseline to postintervention, the video group showed a greater increase (mean change = 0.3, 95% CI: 0.1–0.5; *d* = 0.13) than the control group (mean change = 0.2, 95% CI: 0–0.4; *d* = 0.02; *p* = 0.003). However, these effects were not sustained at the 30‐day follow‐up.

**Figure 2:**
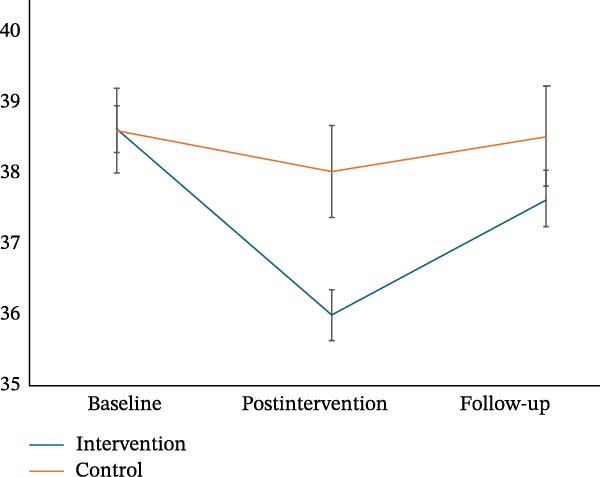
Survivors stigma scores per study arm over time.

Study group outcomes also significantly differed on suicide stigma scores. Figure 2 presents the GEE model results. There were no significant differences between groups at baseline (video intervention: 38.6 ± 10.6; psychoeducational control: 38.6 ± 11.0; independent *t*‐test: *t* = 0.04, *p* = 0.97). A significant group‐by‐time interaction emerged (*χ* ^2^ = 23.0, df = 2, *p* < 0.001). To better understand these differences, we examined changes from baseline to postintervention and from baseline to the 30‐day follow‐up. From baseline to postintervention, the video intervention group showed a greater reduction in stigma scores (mean change = 2.7, 95% CI: 2.0–3.3; Cohen’s *d* = 0.25) compared to the psychoeducational control group (mean change = 0.5, 95% CI: 0–1.2; *d* = 0.05; *p* < 0.001). Effects were sustained at the 30‐day follow‐up, with a reduction of 1.1 points (95% CI: 0.3–1.4; *d* = 0.10) in the video group compared to 0.1 (95% CI: –1.0 to 1.2; *d* = 0) in the control group (*p* = 0.009).

Table 2 presents item‐level changes in the perceptions of personal characteristics associated with SLSs following the intervention. Of the 12 negative traits assessed, 8 (66.7%) showed statistically significant changes from pre‐ to postintervention. Among these, several demonstrated notable shifts toward less stigmatizing perceptions, including “Cowardly” (16% reduction) and “Disconnected” (24% reduction), with smaller but significant decreases for “Immoral” (12%) and “Irresponsible” (12%). Other negative traits, such as “An embarrassment,” “Lost,” and “Stupid,” showed modest improvements that did not reach statistical significance. At the 30‐day follow‐up, effect sizes for negative traits were generally smaller, with only a few—most notably “Cowardly” (38% reduction) and “Disconnected” (10% reduction)—maintaining significant improvement over time. One exception to the overall trend was the trait “Lonely,” which showed increased endorsement following the intervention. Positive traits showed fewer changes. Of the four traits assessed, only “Brave” increased significantly from pre‐ to postintervention (9% increase). From preintervention to follow‐up, “Brave” and “Noble” both showed significant increases.

**Table 2:** Proportion of participants who reversed perceptions of personal characteristics following the intervention.

Items	Agreement with characteristics in reference to suicide survivors								Shifted from stigmatizing to nonstigmatizing perceptions					
	Baseline (*n* = 1012)		Post (*n* = 965)				30 Day FU (*n* = 768)		Baseline to post			Baseline to FU		
	*N*	%	*N*		%		*N*	%	%		*X* ^2^	%		*X* ^2^
Negative characteristics														
Cowardly	309	31	251		26		190	19	**16**		8.9	**38**		9.0
Disconnected	497	49	359		37		337	44	**24**		52.9	**10**		10.4
An embarrassment	695	69	625		65		522	68	**6**		7.1	1		1.5
Immoral	266	26	220		23		190	25	**12**		9.5	4		1.8
Isolated	603	60	572		59		422	55	2		.04	**8**		8.2
Irresponsible	333	33	284		29		241	31	**12**		6.1	6		1.4
Lonely	609	60	618		64		447	58	**−7**		5.4	3		2.7
Lost	692	69	615		64		488	64	**7**		9.9	**7**		6.5
Pathetic	290	29	263		27		200	26	7		1.5	10		1.2
Shallow	278	28	248		26		188	25	7		2.1	11		3.0
Stupid	256	25	210		22		187	24	**12**		7.3	4		0.14
Vengeful	264	26	234		24		191	25	8		2.9	4		0.21
Positive characteristics														
Committed	717	71	668		69		543	71	−2		1.1	0		0.02
Noble	504	50	508		53		428	56	5		1.7	**11**		5.0
Strong	711	70	673		70		534	70	0		0.41	0		0.82
Brave	609	60	632		66		492	64	**9**		11.4	**6**		4.2

*Note:* Higher percentage indicates higher agreement with characteristics. Bold indicates significance at *p* < 0.05 (McNemar’s test).

Table 3 presents item‐level shifts in stigmatizing perceptions following the video intervention. Of the 16 items assessed, 14 (87.5%) showed a statistically significant shift from stigmatizing to nonstigmatizing views at the postintervention timepoint, though the magnitude of the change varied across items. For example, among participants who initially agreed with the statement, “When someone in the family dies by suicide, it probably means the family didn’t do enough to help,” 40% reversed their agreement after the intervention. Similarly, 25% of those who initially endorsed “When someone in the family dies by suicide, it means something is wrong with the family” no longer held that belief. Additional reductions were observed for the items “It is likely that family members of someone who died by suicide suffer from a mental disorder” (24%) and “When someone in the family dies by suicide, the responsibility lies partly with the family” (21%). Other items showing improvement included perceptions of families as cold or unloving (19%) and beliefs that suicide represents a personal failure of the family (19%).

**Table 3:** Proportion of participants who reversed stigmatizing perception following the intervention.

Items	Stigmatizing perceptions toward suicide survivors						Shifted from stigmatizing to nonstigmatizing perceptions			
	Baseline (*n* = 1012)		Post (*n* = 968)		30 Day FU (*n* = 775)		Baseline to post		Baseline to FU	
	*N*	%	*N*	%	*N*	%	%	*X* ^2^	%	*X* ^2^
When someone in the family dies by suicide, it probably means the family didn’t do enough to help them.	478	47	285	28	264	26	**40**	85.4	**45**	38.0
A person whose family member died by suicide is viewed more negatively than others.	489	48	458	47	416	54	2	.50	−13	3.03
Families in which a member died by suicide are usually cold and lacking in love.	254	25	197	20	199	20	**20**	14.1	20	0.44
It is more stressful to talk with family members of people who died by suicide than with other bereaved families.	508	50	539	56	464	60	**−12**	9.7	**−20**	13.4
It is likely that family members of someone who died by suicide suffer from a mental disorder.	334	33	245	25	234	30	**24**	27.5	9	2.1
Talking about suicide with family members of people who died by suicide only causes them more pain.	747	74	679	70	584	75	**5**	4.2	−1	0.23
When someone in the family dies by suicide, the responsibility lies, in part, with the family.	477	47	359	37	325	42	**21**	41.8	**11**	8.2
When someone in the family dies by suicide, it means something is wrong with the family.	326	32	236	24	242	31	**25**	30.9	3	0.64
People whose family member died by suicide are usually more disturbed and less stable than others.	262	26	204	21	200	20	**19**	14.0	23	0.27
Family members of people who died by suicide prefer not to talk about it with others.	779	77	701	72	594	77	**6**	7.7	0	0.00
Suicide in a family indicates that there are many problems within the family and among its members.	389	38	281	29	272	35	**24**	36.0	**8**	4.3
If the family had been more attentive, they could have saved the person who died by suicide.	653	65	505	52	435	56	**20**	65.2	**14**	17.7
Suicide is a sign of personal failure on the part of the family.	312	31	244	25	222	29	**19**	16.9	6	3.6
People whose family member died by suicide are rigid and difficult individuals.	234	23	200	21	196	25	**9**	5.0	−9	1.1
The topic of suicide should not be brought up with people whose family member died by suicide.	719	71	656	68	525	68	**4**	4.9	**4**	5.4
People whose family member died by suicide are vulnerable and fragile.	629	62	550	57	464	60	**8**	11.2	3	0.33

*Note:* Higher percentage indicates higher improvement in stigmatizing attitudes. Bold indicates significance at *p* < 0.05 (McNemar’s test).

However, two items showed increased endorsement of stigmatizing views at postintervention. Agreement with “It is more stressful to talk with family members of people who died by suicide than with other bereaved families” increased by 6%, and the belief that “People whose family member died by suicide are viewed more negatively than others” rose by 13%. While most items showed reductions in stigma—particularly those related to blame, dysfunction and pathology—these two items reflecting social discomfort and perceived judgment saw increases over time, suggesting more nuanced patterns of change across stigma domains.

## 4. Discussion

The present study was designed to address a critical gap in the literature by examining whether brief social contact‐based video narratives of SLSs can reduce public stigma and improve trait impressions by comparing SLS narratives to a written psychoeducational control among 1351 participants. Consistent with our hypotheses, exposure to brief videos of SLS narratives led to meaningful reductions in stigmatizing attitudes toward SLSs and improvements in trait impressions relative to the psychoeducational control. Participants endorsed fewer negative stereotypes such as blame and dysfunction and rated SLSs as warmer and more competent immediately after exposure to the intervention. While overall effect sizes were small, the scalability of the intervention points to the opportunity for the intervention to be implemented as a form of moving the needle on stigma toward SLSs.

Importantly, while the magnitude of effects attenuated over time, both reductions in stigma and improvements in impressions remained detectable at the 1‐month follow‐up, indicating that even brief interventions can exert sustained influence. These findings parallel prior research demonstrating the efficacy of brief video narratives in reducing stigma toward individuals with psychosis [22] and depression [33], thereby extending the evidence base to suicide bereavement—a domain that has remained underexplored despite the documented psychological burden and pervasive stigma faced by survivors [2, 15].

One of the most robust findings was the significant reduction in stigmatizing attitudes toward SLSs following exposure to survivor narratives. Participants expressed lower endorsement of stereotypes that have historically characterized bereaved families as blameworthy, dysfunctional, or socially tainted [15, 19]. This pattern aligns with theoretical models of social contact‐based stigma reduction, which posit that direct or virtual contact with individuals from a stigmatized group humanizes their experience, fosters empathy, and counters negative stereotypes [21]. In the context of suicide bereavement, the authenticity and emotional resonance of first‐person accounts may have disrupted prevailing attributions of responsibility and instead reframed survivors as individuals navigating profound grief under complex social pressures. This is particularly salient given the evidence that stigma toward SLSs is linked to higher distress, depression, and suicidality [2, 20]. By reducing public stigma—even modestly—such interventions may help to alleviate the social isolation and internalized shame that perpetuate poor outcomes among survivors, suggesting that targeted video narratives could play a crucial role in reshaping the social ecology of suicide bereavement.

A second notable finding was the improvement in trait impressions, with participants rating survivors as warmer and more competent after viewing the videos. Item‐level analyses clarified where this shift occurred: immediately postexposure, we observed sizable declines in pejorative attributions—most prominently “Disconnected” (−24%) and “Cowardly” (−16%)—alongside smaller but significant decreases for “Immoral” (−12%) and “Irresponsible” (−12%). By 1 month, effects attenuated overall, yet “Cowardly” (−38% from baseline) and “Disconnected” (−10%) remained significantly improved. This pattern is clinically meaningful because these labels index the very stereotypes that erode survivors’ social standing—moral blame (“cowardly”/“immoral”/“irresponsible”) and relational distancing (“disconnected”). Consistent with evidence that brief social contact‐based videos can shift public attitudes and intentions [22, 33] and with the Papageno effect, whereby narratives emphasizing coping and recovery foster adaptive responses [34], the present results suggest that survivor testimonies functioned as microinterventions of social reappraisal—reframing SLSs from targets of suspicion and moral judgment to relatable individuals worthy of empathy and support. Given the well‐documented barriers to informal and formal help [13, 15, 18], such targeted changes in trait attributions may be a key mechanism by which short, scalable interventions translate into greater willingness to approach, assist, and include SLSs. We also acknowledge that exposure to moralizing items in this scale has the potential to reinforce stigmatizing beliefs; even so, the basis in the lived experience of SLSs in informing this scale supports the notion that these are existing positions worth acknowledging and challenging.

An interesting pattern emerged regarding social discomfort and perceived public judgments. Immediately postintervention, endorsement increased on two items—“more stressful to talk with families bereaved by suicide than other bereaved families” (+6%) and “suicide bereaved families are viewed more negatively than others” (+13%). One potential interpretation is that the vivid survivor contact of the intervention videos may have reduced moralized blame while temporarily heightening awareness of the interpersonal awkwardness faced by SLSs and of their perceptions of society’s negative views of them—phenomena well documented in suicide‐bereavement stigma [14, 15, 18]. If replicated, it may be useful to test light “debrief” prompts that model supportive language and/or booster exposures to consolidate learning [22, 23]. Alternatively, this may be explained by the distinction between stigma toward suicide itself and stigma toward SLSs; while the intervention was effective in reducing stigma toward SLSs, these increases may relate more closely to the stigma and social distance associated with suicide itself. Ultimately, follow‐up studies are needed to clarify the mechanisms (e.g., empathy, identification, and perceived norms), boundary conditions, and behavioral consequences of these nuanced effects across stigma domains.

Although reductions in stigma and improvements in trait impressions persisted at 1 month, their attenuation over time aligns with prior brief‐video trials showing that attitude gains from single, brief exposures tend to weaken without reinforcement [22, 23]. This trajectory is theoretically coherent with social‐cognition accounts: implicit stereotypes and entrenched schemas are resistant to durable change, especially when challenged once and briefly [21]. In suicide bereavement specifically, stigma is culturally embedded in attributions of blame and moral responsibility, which may further impede maintenance [15, 24]. Accordingly, follow‐up strategies should build in repetition and context—for example, scheduled boosters, curricular or organizational embedding, and brief postviewing debriefs that model supportive responses—to consolidate learning and reduce drift back to stigmatizing norms [22, 23]. More broadly, future work should test dose–response and maintenance mechanisms to determine what cadence and format best translate short‐term attitudinal gains into stable, behaviorally meaningful change.

The results of this study should be interpreted considering several important limitations. First, our sample was recruited from an online Israeli panel and limited to Hebrew‐speaking adults, which may restrict generalizability. Online panels typically overrepresent individuals with higher digital literacy and education and underrepresent people at the margins of access. Similarly, the limitation of an Israeli population has implications for the generalizability of the intervention content, as stigma is closely tied to culture. This is true also of the limitation of age, as the range from young to middle‐aged adults may limit generalizability for younger or older individuals. Second, the primary outcomes were self‐reported attitudes (public stigma and trait impressions). Such measures are susceptible to demand and social‐desirability biases and may not translate into behavioral change (e.g., willingness to offer help and actual supportive actions). Incorporating behavioral indices and informant/peer reports would triangulate the effects and strengthen external validity. Third, the stigma instrument toward SLSs demonstrated strong internal consistency, but further validation is warranted: factor structure, convergent/discriminant validity (e.g., with social distance and blame scales), test–retest reliability, and measurement invariance across gender, age, and prior exposure to suicide loss. Without these data, construct interpretations should remain cautious. Finally, we note that reductions in stigma toward SLSs do not equate to reductions in stigma toward suicide itself, which would necessitate a different intervention with a different focus.

### 4.1. Conclusions and Implications

In this randomized study, brief social contact‐based videos featuring SLSs reduced public stigma and improved trait impressions relative to a psychoeducational control; these effects persisted at one month, albeit attenuated. These findings extend the brief‐video anti‐stigma literature [22] to the understudied context of suicide bereavement and point to a low‐cost, scalable complement to postvention. Even modest, durable shifts in attitudes at the population scale are consequential and outline a practical pathway for improving the social climate surrounding SLSs.

Several important implications can be drawn from these results. First, brief social contact‐based videos should be embedded as a scalable postvention component—in schools, workplaces, primary care, and municipal campaigns—due to their immediate and 30‐day reduction effects in stigma toward SLSs, mirroring effects reported in other public‐stigma domains [22, 23]. Second, this approach is clinically meaningful given that public stigma toward families bereaved by suicide—manifesting as shame, blame, avoidance, and secrecy—is associated with greater global distress, depression, self‐harm, and suicidality [13, 15, 35]. Importantly, brief videos can also promote help‐seeking intentions, offering a concrete bridge from attitudinal change to action—especially among youth [33]. Taken together, survivor‐narrative videos represent a low‐cost, widely deployable adjunct to postvention that, when implemented at scale, can help durably improve the everyday social climate surrounding SLSs [36].

## Funding

This research was supported by the Gottesman Fund (Grant 123/2024).

## Disclosure

The funders had no role in study design, data collection, analysis, interpretation, or manuscript preparation.

## Ethics Statement

The study was approved by the Institutional Ethics Committee of the Ruppin Academic Center (Protocol Number 167/2025). All study procedures were conducted in accordance with the Declaration of Helsinki and with the ethical standards for research involving human participants.

## Consent

All participants received detailed written information about the study and provided full informed consent prior to participation. Consent procedures were approved by the ethics committee. Participants could withdraw at any point without penalty.

## Conflicts of Interest

The authors declare no conflicts of interest.

## Supporting Information

Additional supporting information can be found online in the Supporting Information section.

## Supporting information


**Supporting Information 1** Supplement 2: Video links and scripts.

**Supporting Information 2** Supplement 1: Study flow diagram.

## Data Availability

The data that support the findings of this study are available from Yossi Levi‐Belz (corresponding author) upon reasonable request. Deidentified datasets can be shared in accordance with institutional and ethical guidelines.
