# Supplementary material for: Reducing Public Stigma Toward Suicide‐Loss Survivors Through Brief Video Interventions: A Randomized Controlled Trial
Source: Depress Anxiety. 2026 Jul 20;2026:2062450. doi: 10.1155/da/2062450 (PMC13385198; doi:10.1155/da/2062450)
Supplement: Supplementary file 1 — Supporting Information 1 Supplement 2: Video links and scripts. [file DA-2026-2062450-s002.docx]

**Supplement 2- Video links and scripts**

**Video links**

Mother - <https://youtu.be/FJd19jYYXM8>

Father - <https://youtu.be/S2JtJCTXXIE>

Brother - <https://youtu.be/MovAh5uIA-o>

**Scripts**

**Mother**

Hi, I’m Ilana. I’m a mother of three, living in the center of the country, and I’ve worked as a librarian for more than 20 years. Two years ago, I lost my son Yoav. He was 18 when he died by suicide.

From the outside? No one would have believed it. A warm, “normal” family. A sweet boy with lots of friends, just finished high school, getting ready for the army.
But inside? Since Yoav left, everything has been broken. And I’ve been left with endless questions. How did I not see it? Why didn’t he come and tell me? Where did I go wrong as a mother?

For months I walked around with shame and guilt. I didn’t tell anyone how he died—I just said he passed away suddenly. I was afraid people would judge us. Think something was “wrong” with our family. That we had failed him. That we could have stopped it but didn’t do enough. That we didn’t protect him.

And the people around me—friends, colleagues—they didn’t ask. Maybe they were afraid to hurt me, or afraid I would fall apart. Maybe they just didn’t know what to say. I pulled away. I didn’t want pity. I felt so alone.

Then, at his one-year memorial, I suddenly heard myself say the word “suicide” out loud.
And something inside me released.

Since then, I’ve started to talk. With friends. With my family. With other mothers who also lost a child. It wasn’t easy, but I realized I had nothing to be ashamed of.

Today I understand, it was depression, the mental illness he suffered from, that led Yoav to take his life. Not a fight, not a crisis, and not because of our parenting. In that moment, the illness won. And this happens—even in good families.

The pain is still here, and it always will be. But I don’t face it alone anymore. And that has allowed me to keep going on my journey.

**Father script**

Hi, I’m Amit. I’m a father of three, and I’ve been an engineer for almost twenty years. Two years ago, I lost my son Alon—he died by suicide at the age of 27.

From the outside, I probably looked fine, like I had moved on. But inside, everything was shattered.
I couldn’t stop asking myself the hardest questions: How did I not see it? How did I miss it? Maybe I was just a bad father?

I kept replaying our conversations, remembering every time he seemed tired or sad, and blaming myself for not listening more, for not asking enough.
The guilt was crushing. I felt it toward myself and toward others, like a weight on my chest. I couldn’t sleep.

I stopped talking about Alon. Even with my other kids, I stayed silent. As if not saying it out loud meant it didn’t happen.
And deep down, I felt I had betrayed him, that I hadn’t protected him enough. That was supposed to be my job.

Then, a few months later, my wife convinced me to attend a lecture on suicide prevention.
I sat in the back, barely paying attention, until the speaker said:
“Even if you did everything, sometimes it’s still not enough. Because the illness, depression, was stronger.”

Those words broke me. For the first time since Alon was gone, I cried.

And from then on, slowly, I began to talk—with my wife, with my kids, with a friend.
I joined a support group for bereaved parents.
It doesn’t heal the pain, but it changes the way you carry it.

Today I understand: Alon didn’t leave because he didn’t love us, he left because the pain was unbearable.
This can happen, even in the most loving families.
The grief is still with me - it’s part of who I am - but now I don’t carry it alone.

**Brother script**

Hi, I’m Omer, Alon’s brother.
Alon died by suicide two years ago. He was 27.

Honestly? It was such a shock. I never thought it could happen.
Yes, he was quiet and kept to himself, but I always thought he was managing. I just didn’t realize how much he was suffering.

After it happened, most people talked about my parents, about how hard it must be for them.
And me… I felt invisible. Like it didn’t happen to me too. Like if you’re not the parent, then it’s not “really” yours.

But the truth is, Alon was part of me. He was my brother, my friend.
The pain burns inside me. It’s such a huge loss.

For a long time, I kept asking myself if I did enough. If there was something I could have done to stop it.
Why didn’t I notice? Why didn’t I ask one more question?
And I felt like some people thought it happened because of us—as if it was the family’s fault.
That just added even more pain to what we were already carrying.

But here’s what I know now: depression is not anyone’s failure.
It’s an illness. A serious illness.
And sometimes, even when there’s love and even when you do everything right, it’s still not enough.

At first I didn’t want to talk. I kept everything inside.
But eventually, it was just too much.
I started talking, to a friend, then to family.
It didn’t erase the pain, but it really helped.

Today I know this: even if he didn’t ask for help, it doesn’t mean he didn’t need it.
And Alon didn’t leave because he gave up on us.
He just couldn’t see another way.

**Psychoeducational Control Text**

Suicide stigma refers to negative attitudes, misconceptions, and prejudices toward people who are struggling with suicidal thoughts or who have lost a loved one to suicide. This stigma can manifest itself on a public level — for example, in the belief that suicidality is a manifestation of personal weakness or moral failure — as well as on an internal level, when a person internalizes negative attitudes toward themselves because of their thoughts or experiences. This social and personal stigma can prevent people from seeking mental health help, exacerbate feelings of shame and guilt, and lead to avoidance of open discussion about the issue. Research indicates that stigma is one of the main barriers to treatment and prevention in the field of suicide. Family members of people who have committed suicide may experience social stigma and sometimes even blame themselves, which makes the process of coping and grieving more difficult.
