# Supplementary material for: Reducing Public Stigma Toward Suicide‐Loss Survivors Through Brief Video Interventions: A Randomized Controlled Trial
Source: Depress Anxiety. 2026 Jul 20;2026:2062450. doi: 10.1155/da/2062450 (PMC13385198; doi:10.1155/da/2062450)
Supplement: Supplementary file 2 — Supporting Information 2 Supplement 1: Study flow diagram. [file DA-2026-2062450-s001.docx]

**Supplement 1.** Flow Diagram

Note: 187 participants (12%) were excluded due to failing validity checks, including attention checks, invalid responses to questions, or being identified as bots.
